# Biocompatibility of a Novel Cyanoacrylate Based Tissue Adhesive: Cytotoxicity and Biochemical Property Evaluation

**DOI:** 10.1371/journal.pone.0079761

**Published:** 2013-11-22

**Authors:** Young Ju Lee, Gyeong Bok Jung, Samjin Choi, Gihyun Lee, Ji Hye Kim, Ho Sung Son, Hyunsu Bae, Hun-Kuk Park

**Affiliations:** 1 Department of Biomedical Engineering and Healthcare Industry Research Institute, Kyung Hee University, Seoul, Korea; 2 Department of Medical Engineering, Graduate School Kyung Hee University, Seoul, Korea; 3 Department of Physiology, College of Korean Medicine, Kyung Hee University, Seoul, Korea; 4 Department of Thoracic and Cardiovascular Surgery, College of Medicine, Korea University, Seoul, Korea; Indian Institute of Toxicology Reserach, India

## Abstract

Cyanoacrylate (CA) is most widely used as a medical and commercial tissue adhesive because of easier wound closure, good cosmetic results and little discomfort. But, CA-based tissue adhesives have some limitations including the release of cytotoxic chemicals during biodegradation. In previous study, we made prepolymerized allyl 2-CA (PACA) based tissue adhesive, resulting in longer chain structure. In this study, we investigated a biocompatibility of PACA as alternative tissue adhesive for medical application, comparing with that of Dermabond® as commercial tissue adhesive. The biocompatibility of PACA was evaluated for short-term (24 hr) and long-term (3 and 7 days) using conventional cytotoxicity (WST, neutral red, LIVE/DEAD and TUNEL) assays, hematoxylin-eosin (H&E) and Masson trichrome (MT) staining. Besides we examined the biochemical changes in cells and DNA induced by PACA and Dermabond® utilizing Raman spectroscopy which could observe the denaturation and conformational changes in protein, as well as disintegration of the DNA/RNA by cell death. In particular, we analyzed Raman spectrum using the multivariate statistical methods including principal component analysis (PCA) and support vector machine (SVM). As a result, PACA and Dermabond® tissue adhesive treated cells and tissues showed no difference of the cell viability values, histological analysis and Raman spectral intensity. Also, the classification analysis by means of PCA-SVM classifier could not discriminate the difference between the PACA and Dermabond® treated cells and DNA. Therefore we suggest that novel PACA might be useful as potential tissue adhesive with effective biocompatibility.

## Introduction

Tissue adhesive is an attractive alternative to the traditional wound closure techniques such as sutures and staples. It should allow rapid adhesion, close apposition of wound edges, and maintenance of strong wound cover for a sufficient time [1]. Good tissue adhesives should be simple, effective, safe, rapid, painless and biodegradable with minimal tissue toxicity. They should also result in an optimal cosmetic appearance of the resultant scar [2], [3].

Cyanoacrylates (CA) possess some of these properties and can be applied in medicine with good cosmetic outcomes. The commercial CA tissue adhesives for medical applications are octyl-2-CA (Dermabond®, Johnson & Johnson/Ethicon, Somerville, NJ) and n-butyl-CA (Histoacryl®, B. Braun, Melsungen, Germany) which are longer chain derivatives. The length of the alkyl chain is important because the toxicity can be reduced with increased carbon number in the alkyl chain [4]–[7]. Medical applications must be non-toxic, no harmful side effects. Therefore, assessment of the cell viability and cytotocixity is a necessary step in the evaluation of biocompatibility [8]. In addition, in order to avoid overestimation or underestimation of the toxicity of biomaterials, more than one assay should be used to determine cytotoxicity assay, as this would increase the reliability of the results obtained [9], [10].

Raman spectroscopy is a powerful analytical technique that is rapid, label-free, non-invasive, non-destructive and has high sensitivity, which can be used for the analysis of biological samples [11]. This is a well-established tool used in many research fields to directly investigate the molecular compositions and structures of the biological samples [12], [13]. This technology represents highly multiplexed biochemical information of DNA, RNA, proteins, and lipid content as well as conformation of the living cell by spectral shape or intensity [14]. Raman spectroscopy has been applied to analyze the toxic effects of polymeric nanoparticles and gold nanoparticles. It is well-suited technique to uptake studies of nanoparticles into cells, as well as the cytotoxicity study for drug delivery [15], [16]. Yao et al reported the potential of Raman spectroscopy as distinguishing between a single apoptotic cell and carcinoma cell for monitoring apoptosis, evaluating the efficacy of anti-cancer drug induced apoptosis in gastric carcinoma cells [17]. The cytotoxic effects of toxic agent on living cells could be evaluated from Raman spectra of biochemical change related to cell death [18].

Previously, we reported that partial pre-polymerization of allyl 2-cyanoacrylate (PACA) causes longer chain structure leading to improvement the biocompatibility of common CA [19]. In this study, we evaluated and compared *in vitro* and *in vivo* biocompatibility of the PACA and commercial CA tissue adhesive Dermabond®. The cytotoxicity on the PACA and Dermabond® was tested with direct and indirect contact for time course on fibroblast cell culture, and changes in the biochemical property at a molecular level after the exposure to tissue adhesives using Raman spectroscopy with multivariate statistical methods including principal component analysis (PCA) and support vector machine (SVM). These results were discussed the change of protein and DNA related to the cell death. The biocompatibility *in vivo* on the PACA and Dermabond® was confirmed by histological analysis of animal dermal tissues.

## Results

### 2.1. Cytotoxicity of tissue adhesives assessment for short time using four different assays

**Figure 1:**
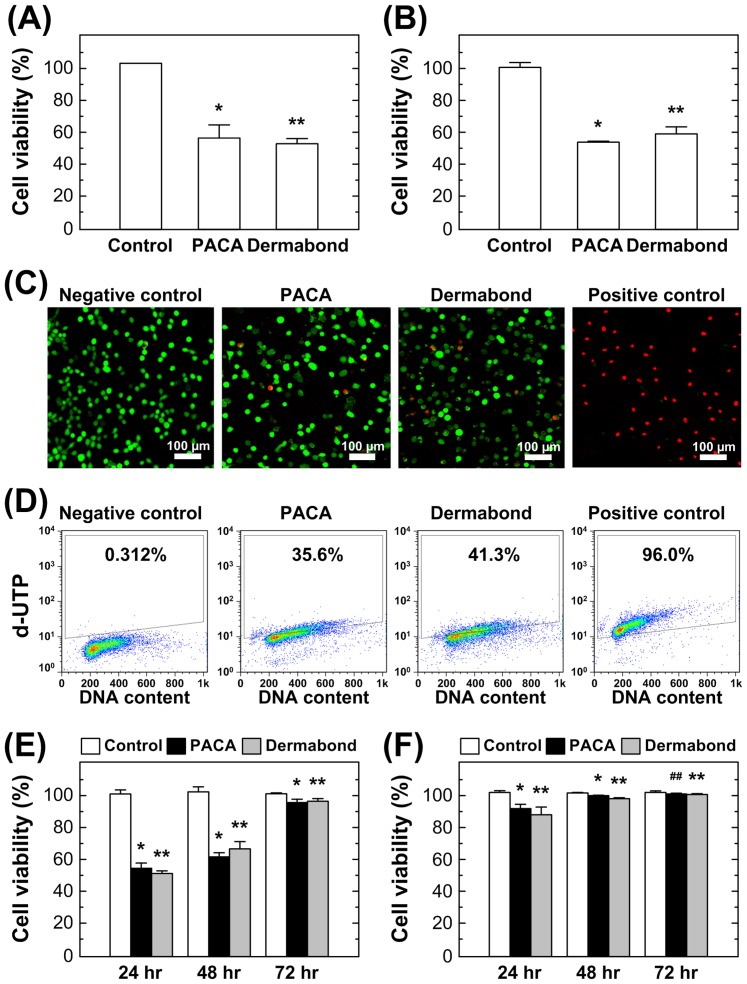
Evaluation of cytotoxicity after tissue adhesives treatments for short-time using four conventional assays. Mouse L929 fibroblasts (1×10^5^ cells) were seeded in a 12 well plates. The cells were incubated control and treated with two tissue adhesives (PACA and Dermabond®) and then further incubated for 24 hrs. Cell viability was analyzed using the (A) WST assay, (B) NR assay, (C) LIVE/DEAD viability/cytotoxicity assay, Scale bars = 100 µm, (D) TUNEL assay. *P<0.05 vs. control (n = 6 pairs). **P>0.05 vs. PACA (n = 6 pairs). **Evaluation of cytotoxicity after tissue adhesives for time course on fibroblast cell culture.** Two tissue adhesive were treated with (E) direct contact and (F) indirect contact for 24 hr, 48 hr, and 72 hr. Cell viability was analyzed using the WST assay. *P<0.05, ^#^P>0.05 vs. control (n = 4 pairs). **P>0.05 vs. PACA (n = 4 pairs).

Mouse fibroblast L929 cells were treated with same dose of the PACA and Dermabond® tissue adhesives (5 µl/10^5^ cells) for 24 hr by direct contact. Cytotoxicity was assessed by the Water-soluble tetrazolium salt (WST), Neutral red (NR), LIVE/DEAD and TUNEL assays. The results were observed in experiments performed in triplicate. Both the PACA and Dermabond® tissue adhesives had similar cell viability values compared to the controls (Fig. 1A and 1B). The WST and NR assays showed 54–57% cell viability for the cells treated with the PACA tissue adhesive and 53–57% cell viability for the cells treated with the Dermabond® tissue adhesive. This result was confirmed by LIVE/DEAD viability/cytotoxicity assay using confocal microscopy. Representative images were selected from the results of one set experiment among three experiments. There was no difference in cell viability between the PACA and Dermabond® tissue adhesive treated cells (Fig. 1C). Fig. 1D shows the results of the TUNEL assay, apoptotic cell detections of TdT-Fluor in the control and two tissue adhesives treatment were compared to confirm apoptosis and quantify cell death. Dermabond® tissue adhesive treatment induced small scale DNA fragmentation compared to the PACA tissue adhesive.

### 2.2. Cytotoxicity of tissue adhesives assessment for time course using direct and indirect contact test

To evaluate the cytotoxicity for time course, the cells were seeded to a 12 well plate and maintained at 37°C for 24 hr. The PACA and Dermabond® were added directly to the center of each well for times courses (24 hr, 48 hr and 72 hr) and cell viability was assessed by WST assay. Cell viability (Fig. 1E) was increased with the time and all the cells were survived at 72 hr. Both the PACA (54.4%, 61.1% and 94.2%), and Dermabond® (50.8, 66.1% and 95.3%) had similar cell viability values on the time course (24–72 hr). In the indirect contact method, PACA and Dermabond® were allowed to medium for 24 hr at 37°C before cell treatment. Mouse fibroblast L929 cells were exposed elution medium of PACA and Dermabond® for time courses. Cell viability (Fig. 1F) showed more than 80% cell viability on the time course. Indirect contact method showed no cytotoxicity both PACA and Dermabond®.

### 2.3. Evaluation of *in vivo* biocompatibility from incision rat model

**Figure 2:**
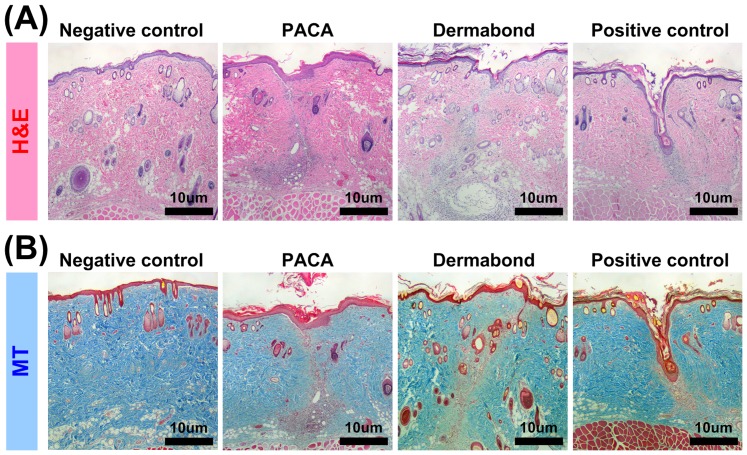
Evaluation of *in vivo* biocompatibility from incision rat model. Histological analysis of Normal (negative), PACA-treated, Dermabond®–treated and untreated (positive) Sprague–Dawley rats at day 7 by (A) H&E staining and (B) MT staining. Scale bars = 10 µm.

To evaluate the *in vivo* biocompatibility, we performed histological analysis. Figure 2 shows the representative H&E and MT staining images at day 7 after incision and PACA or Dermabond® treatments. The tissue adhesive-treated group showed the healing of incision area, whereas the non-treated group was not sealed. Also, both tissue adhesive treatment groups showed the similar distribution of collagen in the dermal layer. However PACA group showed less pathologic inflammation than Dermabond® group.

### 2.4. Effect of PACA and Dermabond® tissue adhesives on Raman spectral of cells and DNA

**Figure 3:**
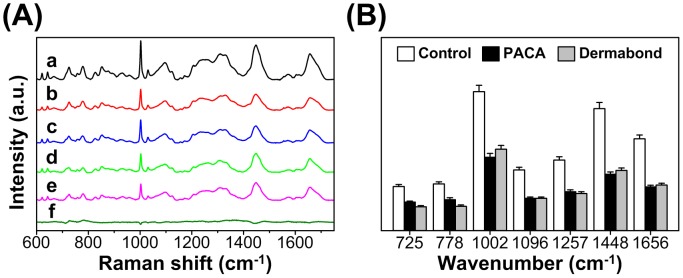
Evaluation of cytotoxicity after tissue adhesives treatments using Raman spectroscopy. (A) Averaged Raman spectra of L929 cells (a: control, b: PACA, c: Dermabond®) and the spectral differences of control and treated cells (d: control-PACA, **e:** control-Dermabond®, f: PACA-Dermabond®). (B) Relative intensities of the Raman peaks for control and treated cells with two tissue adhesives (PACA and Dermabond®).

Fig. 3A shows the mean Raman spectra of the control and two adhesive treated cells (a: control group, b: PACA group, c: Dermabond® group) and the mean spectral differences between the control and tissue adhesives (d: control-PACA group, e: control-Dermabond® group, f: PACA-Dermabond® group). It is clear that all Raman spectra were significantly decreased when the cells were treated with PACA and Dermabond® as compared to control cells. Raman intensity of the PACA and Dermabond® treated cells is almost similar. We selected some specific Raman spectra of compared the changes in their spectral intensities. The changes of spectra at 725 cm^−1^ (adenine), 778 cm^−1^ (cytosine/thymine ring breathing of the DNA/RNA), 1002 cm^−1^ (symmetric ring stretching phenylalanine), 1096 cm^−1^ (phosphodioxy groups, PO_2_^−^, of the DNA backbone) [24], 1257 cm^−1^ (amide III β-sheet of proteins), 1656 cm^−1^ (amide I α-helix of proteins) are from cells (Table 1). The main spectra intensity changes related to the proteins can be observed at 1002 cm^−1^, 1257 cm^−1^, and 1656 cm^−1^ [17], [18], [23]. The Raman spectral intensities of tissue adhesive treated cells were significant decrease in the protein vibrations at 1002 cm^−1^ (43%), 1257 cm^−1^ (45%) and 1656 cm^−1^ (50%). The reduction of Raman spectral intensity corresponding to DNA/RNA vibrations at 725 cm^−1^ (36%) 778 cm^−1^ (33%) and 1096 cm^−1^ (46%) (Fig. 3B).

**Table 1:** Peak assignment for Raman spectra of L929 cells.

Peak (cm^−1^)	Assignment			
	DNA/RNA	Proteins	Lipids	Carbohydrates
1656		Amide I, α-helix	C = C stretching	
1604		C = C Phenylalanine, Tyrosine		
1585	Guanine, Adenine			
1470	Guanine			
1448	Guanine, Adenine	CH deformation	CH deformation	CH deformation
1257	Thymine, Adenine	Amide III, β-sheet	= CH bend	
1245		Amide III, β-sheet		
1207		C-C_6_H_5_ stretching Phenylalanine, Tryptophan		
1156	Deoxyribose, Phosphate	C-C/C-N stretching		
1096	PO_2_^−^ stretching		Chain C-C stretching	C-O, C-C stretching
1002		Symmetric Ring breathing Phenylalanine		
932		C-C backbone stretching α-helix		C-O-C glycos
911	Deoxyribose			
877			C-C-N^+^ symmetric stretching	C-O-C ring
853		Ring breathing Tyrosine		
828	O-P-O asymmetric stretching	Ring breathing Tyrosine		
778	Uracil, Cytosine, Thymine ring breathing			
725	Adenine			
643		C-C twist Tyrosine		
621		C-C twist Phenylalanine		

**Figure 4:**
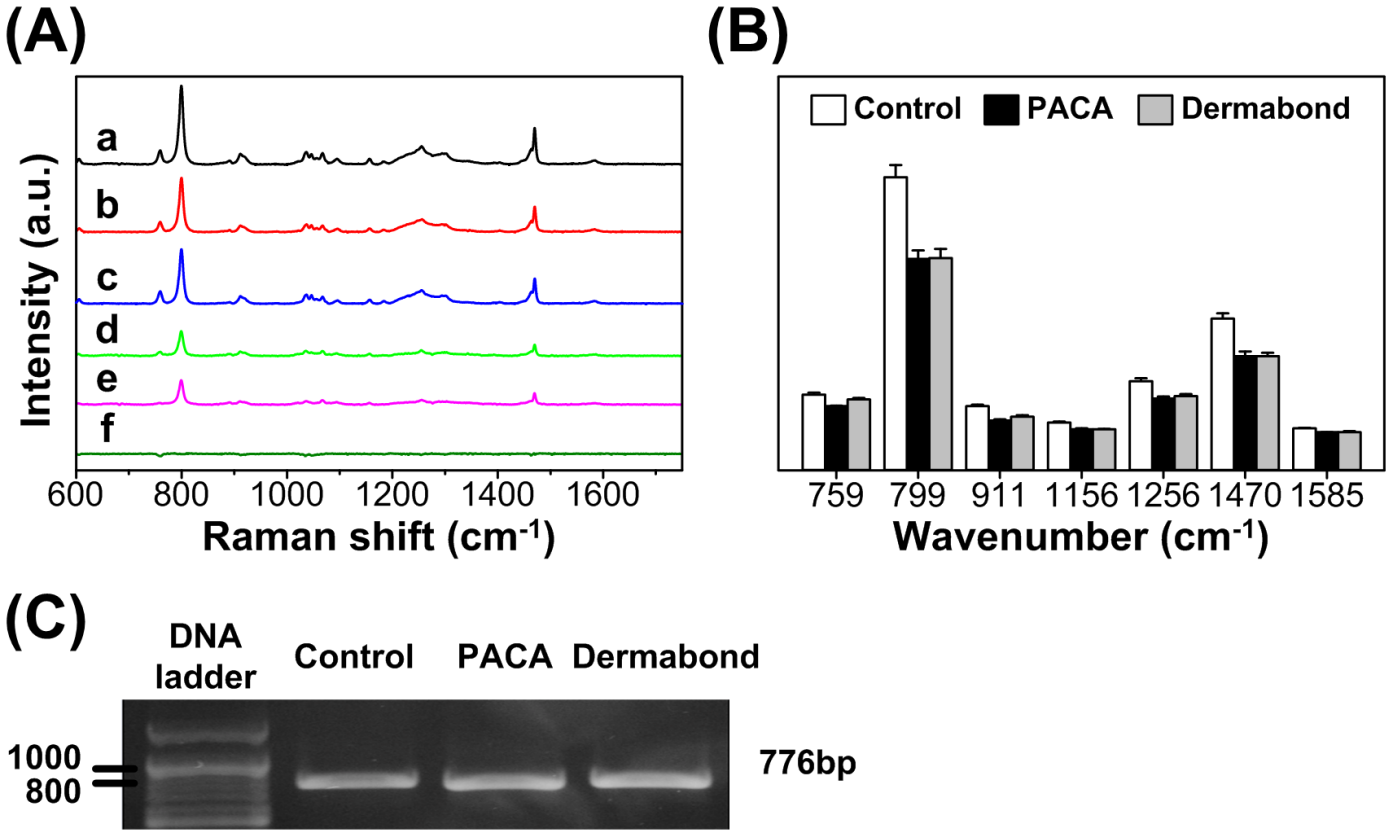
Raman spectroscopy to evaluate cytotoxic changes in response to effects of tissue adhesive on the DNA. (A) Averaged Raman spectra of DNA (a: control, b: PACA, c: Dermabond®) and the spectral differences of control and treated DNA (d: control-PACA, e: control-Dermabond®, f: PACA-Dermabond®). (B) Relative intensities of the Raman peaks for control DNA and treated DNA with two tissue adhesive (PACA and Dermabond®). (C) PCR amplification of the 776 bp β-actin using same dose of DNA samples (Control, PACA and Dermabond®).

Binding with cellular DNA is a crucial step in the mechanism of cytotoxicity. In order to assess the effect of tissue adhesive treatment on cellular DNA spectral signatures, we investigated the spectra of the genomic DNA. DNA was isolated from the cells to minimize the interfering signals from cytoplasm. Raman spectra of each DNA were obtained from an average of 15 spots with same dose of DNA samples. Polymerase chain reaction (PCR) products β-actin was used as the internal control (Fig. 4C). Fig. 3A shows Raman spectra obtained from control DNA and two tissue adhesive treated DNA, a: control group, b: PACA group and c: Dermabond® group and the mean spectral differences between the control DNA and tissue adhesives treated DNA, d: control-PACA group, e: control-Dermabond® group, f: PACA-Dermabond® group. All Raman spectral intensity decreased compared to the control DNA (Fig. 4A and B). The changes of spectra at 759 cm^−1^(ring breathing tyrosine) 799 cm^−1^ (P-O-C symmetric stretching), 911 cm^−1^ (deoxyribose), 1156 cm^−1^ (deoxyribose and phosphate) 1257 cm^−1^ (thymine and adenine), 1470 cm^−1^ (guanine) and 1585 cm^−1^ (guanine and adenine) were from DNA (Table 1). The reduction of Raman spectral intensity at 799 cm^−1^ (30%), 1257 cm^−1^ (26%) and 1470 cm^−1^ (30%) (Fig. 4B). We found no differences in the two adhesive for the cytotoxicity effect.

### 2.5. PCA-SVM classifiers for evaluating biocompatibility of PACA and Dermabond® tissue adhesives on Raman spectral of cells and DNA

**Figure 5:**
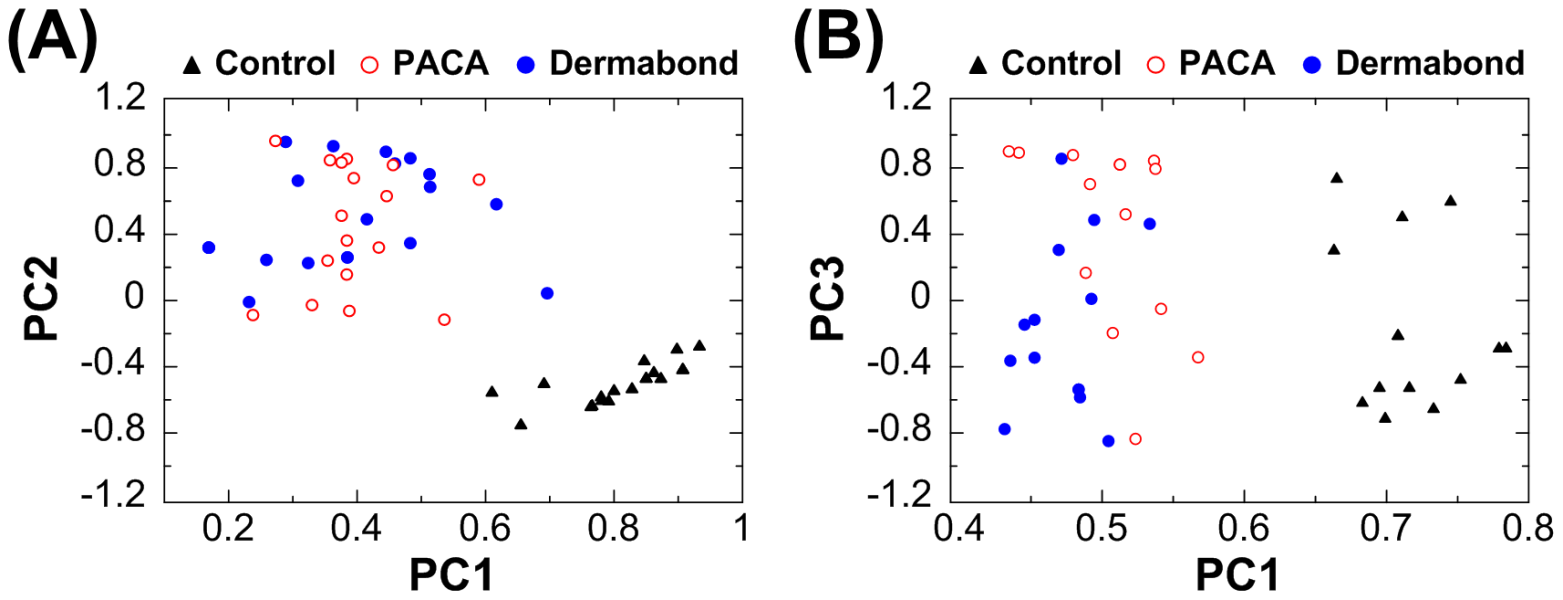
PCA-SVM scores of the control and two tissue adhesive-treated (A) cells and (B) DNA. The SVM classifier was used with the linear discriminant function.

To estimate the cytotoxicity of two tissue adhesives, the PCA-SVM analysis was applied to Raman spectra of cells and DNA. The first step for the discrimination analysis was to determine the most relevant PC in PCA analysis of Raman spectra for cells and DNA, prior to input for SVM analysis. PCA analysis was performed at cytotoxicity associated with six or principal wavelengths of Raman spectra for each group. Only two PCs (more than 99% of the variance) were retained for SVM analysis; PC1 and PC2 for cells and PC1 and PC3 for DNA. The SVM classifier with linear discriminant function was used to discriminate between cytotoxic and non-cytotoxic tissue adhesives by means of the capability of Raman spectroscopy. For Raman spectroscopy based cytotoxicity assessment in cells (Fig. 5A), the tissue adhesive treated cells showed the significant difference with 97.14% sensitivity, 94.44% specificity and 96.23% accuracy compared to the controls (n = 18, Table 2). However, the proposed SVM classifier with linear kernel could not discriminate between the PACA and Dermabond® treated cells, due to high correlation between two tissue adhesives. For Raman spectroscopy-based cytotoxicity assessment in DNA (Fig. 5B), the tissue adhesive treated DNA showed the significant difference with 100% sensitivity, 100% specificity and 100% accuracy compared to the controls (n = 14, Table 2). Similar to the cells there was no significant difference between the PACA and Dermabond® treated DNA samples.

**Table 2:** Classification rate of the proposed PCA-SVM classifiers for evaluating biocompatibility of the PACA and Dermabond® tissue adhesives on the Raman spectral of cells and DNA.*

Specimen	Total No.	TP	FP	TN	FN	SEN	SPE	ACC
Cell	53	34	1	17	1	97.14%	94.44%	96.23%
DNA	42	28	0	14	0	100%	100%	100%

* TP; true positive, FP; false positive, TN; true negative, FN; false negative, SEN; sensitivity, SPE; specificity, ACC; accuracy.

## Discussion

The cytotoxicity assessment is the first process for examining the biocompatibility of biomaterials. Particularly, it is essential for developing new biomedical materials that modulate cell viability and to identify potential cytotoxic side effects of biomedical devices. This study was designed to investigate the cytotoxicity of new pre-polymerized of CA-based tissue adhesive (PACA) using a direct and indirect contact test *in vitro* cell culture. Direct contact testing of novel biomaterials with *in vitro* cell cultures may provide an early indicator of potential problems *in vivo* [20]. The tissue adhesives release toxic substances, so cell cytotoxicity was tested by indirect contact. Mouse L929 fibroblasts which are the main cellular component of dense connective tissues were used for cytotoxicity evaluation of tissue adhesive. Furthermore, L929 fibroblasts are the cells recommended by the international standard ISO-10993-5 for *in vitro* biocompatibility evaluations.

We comparatively assessed *in vitro* and *in vivo* cytotoxicity of new tissue adhesive PACA and commercially available tissue adhesive Dermabond®. The four cytotoxicity assays were employed to assess the toxicity of tissue adhesives *in vitro* at short time and showed that the PACA and Dermabond® are similar cytotoxicity (Fig. 1A–D). Although the Dermabond® tissue adhesive treatment induced small scale DNA fragmentation from apoptotic cell death, the marked incorporation of FITC, compare to the PACA tissue adhesive (Fig. 1D). Also, the direct and indirect contact tests with the time course showed similar cell survival between PACA and Dermabond® (Fig. 1E and F). PACA tissue adhesive showed the most effective cell viability. The finding of PACA tissue adhesive suggests that the polymerization process by heat treatment could decrease the cell cytotoxicity [21]. *In vivo* histological analysis was performed to assess long-term biocompatibility (Fig. 2). The PACA and Dermabond® treatment group were enhanced wound healing than the positive control group, whereas the PACA group showed less inflammation compared to the Dermabond® group. These finding suggests that the PACA tissue adhesive led to superior skin tissue closure, less inflammation, and less foreign body reaction compared to the Dermabond®.

Raman spectroscopy technique is based on the inelastic scattering of laser photons by the molecules of the biological samples. Thus, Raman spectrum represents a molecular fingerprint of their composition. Some studies evaluated the cytotoxic changes in response to adverse effects of chemicals agents on cells using Raman spectroscopy. This technique has the advantage of overcoming the limitations of conventional cytotoxicity assays for identification and quantification of chemicals agents [14], [22]. Furthermore, Raman spectroscopy could distinguish the different stages of the cell cycle from the cells as well as identify unique DNA and protein peaks of dead cells in the culture within only few minutes [23], [24].

In this study, Raman spectroscopy was used to detect and identify the change in cellular components including protein, DNA/RNA, lipid and carbohydrates related to the cytotoxicity by tissue adhesive treatment. Individual L929 cells showed specific Raman spectra of cellular components by tissue adhesives treatment (Table 1). The spectra of cells treated with PACA and Dermabond® tissue adhesives showed large decrease in the spectra corresponding to DNA/RNA vibrations at 725 cm^−1^, 778 cm^−1^, 799 cm^−1^, 1257 cm^−1^, 1470 cm^−1^, and 1096 cm^−1^ and significant decrease intensity corresponding to protein vibrations at 1002 cm^−1^, 1257 cm^−1^, and 1656 cm^−1^.

The most of important the decrease of the spectra was correlated with positions to DNA/RNA including DNA phosphodioxy group PO_2_^−^ stretching, DNA backbone O-P-O asymmetric stretching and DNA bases cytosine, thymine and adenine (Table 1). The phosphate group of the phosphodioxy PO_2_^−^ stretching is sensitive to DNA structure [25]. A decrease in the spectral intensity of DNA structure, DNA backbone and DNA bases is likely to be affected by DNA strand breaks and nuclear fragmentation during the cell death [18]. These changes of intensity might be caused by the destruction of the ring structure, indicating disintegration of the DNA/RNA related to the cell death [26]–[28].

Besides the decrease in the Raman spectra of DNA/RNA, the Raman spectra of cells indicated changes in protein vibrations. The spectral intensity at 1002 cm^−1^ corresponding to the ring breathing of phenylalanine showed 43% reduction and that in the spectra at 1257 cm^−1^ corresponding to cell death showed 45% reduction. A decrease in the protein content in dying cells followed by the induction of the apoptosis proteins for cleavage and slicing is suggested by the decrease in most Raman spectra of proteins including phenylalanine (1002 cm^−1^ and 621 cm^−1^), tyrosine (853 cm^−1^) and C-C backbone stretching α-helix (932 cm^−1^) [26]. The spectral intensity at 1656 cm^−1^ corresponding to amide I α-helix showed 50% reduction. This spectra was observed in Raman spectra of dead cells, indicating conformational changes of proteins [18], [26]–[28]. A decrease in Raman spectra corresponding to proteins might indicate denaturation and conformational changes in proteins related to the cell death. This change involves with molecular mechanisms of membrane proteins signaling with the biochemical changes of the nucleus [18]. We suggest that the spectral regions of most dramatic changes, 725 cm^−1^, 778 cm^−1^, 1096 cm^−1^, 1002 cm^−1^, 1257 cm^−1^ and 1656 cm^−1^ could be used a maker of the cell death for cytotoxicity study.

PCA is a multivariate statistical technique used in bioinformatics. It analyzes a data matrix that represents the observations taken by several intercorrelated variables. The main goal of PCA technique is to extract dominant information from the complicated data matrix and to express the features as a set of new orthogonal variables called a PC. We compared the cytotoxicity between the control and tissue adhesive treated cells and DNA by combining Raman spectral information with PCA-SVM classifier as bioinformatics technique [28], [29]. PCA-SVM analysis with linear discriminant function led to more than 99% of the variance from both cells and DNA. A two-dimensional plot (PC1, PC2) for cells and (PC1, PC3) for DNA clearly showed the no difference between PACA and Dermabond® tissue adhesives (Fig. 5). The result suggests that PACA tissue adhesive showed almost similar cytotoxicity effect on cells and DNA compared to commercial Dermabond® tissue adhesive.

In summary, we have presented the biocompatibility of PACA tissue adhesive for clinical application using conventional cytotoxicity (WST, NR, LIVE/DEAD and TUNEL) assays and Raman spectroscopy with PCA-SVM analysis. Additionally, we evaluated the changes in biochemical properties using Raman spectroscopy. The Raman spectra indicated significant changes in biochemical properties related to nucleic acids and proteins by the tissue adhesive treatment. The reduction of Raman intensity is responsible for conformational changes of proteins, structure change of DNA and degradation of DNA related to the cell death. The cytotoxicity of PACA tissue adhesive in all methods does not suffer by comparison with commercial tissue adhesive Dermabond®. Therefore, these finding indicate that PACA tissue adhesive could be useful an alternative tissue adhesive for skin wound sealing.

## Materials and Methods

### 4.1. Cell culture

Mouse L929 fibroblasts (NCTC clone 929 Korea Cell Line Bank,Seoul, South Korea) were grown at 37°C in a 5% CO_2_ incubator in RPMI 1640 (GIBCO, Grand Island, NY) supplemented with 300 mg/ml L-glutamate, 25 mM hydroxyethyl piperazineethanesulfonic acid (HEPES), 25 mM NaHCO_3_, 10% fetal bovine serum (FBS), 50 µg/µl gentamicin, 500 U/ml penicillin and 500 mg/ml streptomycin. To preparation of PACA, ACA (920; Robinson St. Pottstown, PA, USA) was heated at 150°C for 40 min in vacuum vial (10 ml/vial) and then cooled to 0°C.

### 4.2. WST assay

Mouse L929 fibroblasts (1×10^5^ cells) were seeded in a 12 well plates and incubated for 24 hr. Half maximal inhibitory concentration (IC_50_) for Dermabond® is 5 µl/10^5^ cells. The cells were incubated in direct contact with PACA and Dermabond® (5 µl/10^5^ cells) for 24 hr at 37°C. Cell cytotoxicity was determined using a Cell counting-8 kit (Sigma Aldrich.St. Louis, MO, USA). OD_450_ was recorded utilizing the Synergy HT multi-mode microplate instrument (BioTek, Winooski, VT, USA) and converted to cell viability percentage as compared to the control. To examine the time course of cell cytotoxicity, the cells were incubated in direct contact with PACA and Dermabond® for 24 hr, 48 hr and 72 hr. The cells were determined for cytotoxicity assessment using Cell counting-8 kit.

### 4.3. NR assay

The NR assay determines the accumulation of the NR dye (Sigma Aldrich. St. Louis, MO, USA) in lysosomes of viable cells. The cells were incubated for 2–3 hr with culture medium including 0.005% NR following the exposure to PACA and Dermabond®. The cells were then washed with PBS and 150 µl desorbing fixative solution (EtOH/AcCOOH, 50%/1%) was added followed by gentle shaking for 10 min so that complete dissolution was achieved. OD_595_ was recorded using the multi-microplate reader.

### 4.4. LIVE/DEAD viability/cytotoxicity assay

Two color fluorescent cell cytotoxicity assay (LIVE/DEAD viability/cytotoxicity kit; Molecular Probes, Eugene, OR, USA) was used to confirm the results obtained from the WST assay. After the exposure of 24 hr tissue adhesives, the cells were incubated with a mixture of 8 µM ethidium homodimer (EtdD-1; red color) and 2 µM calcein acetoxymethyl (calcein-AM; green color) in PBS for 30–45 min at room temperature (RT). Stained samples were washed with PBS. Images were collected using a Ziess LSM-700 confocal microscopy system (Thornwood, NY, USA). The numbers of viable (green) and non-viable (red) cells were counted automatically with Image J software (National Institutes of Health, Bethesda, MD, USA). All experiments were performed in triplicate.

### 4.5. TUNEL assay

TUNEL assay was performed in PACA and Dermabond® treated L929 cells using a DeadEnd fluorometric TUNEL System. The cells were immersed the fixed in 4% paraformaldehyde in PBS at 4°C and then were permeabilized with a 70% ice-cold ethanol at −20°C for 5 min. The cells was centrifuged and washed two times with PBS. After centrifugation, resuspend with rTdT reaction mix. Incubate in 37°C water bath for 60 min, protecting from direct light exposure. The reaction terminated by 20 mM ethylenediaminetetraacetic acid (EDTA) and washed two times with 0.1% Triton X-100 solution in phosphate buffered saline (PBS) containing 5 mg/ml BSA. Resuspend the cell pellet in 7-Aminoactinomycin D (7**-**AAD, Sigma Aldrich, St. Louis, MO, USA) solution at RT for 30 min in the dark. Cells were analyzed by FACSCalibur using the CellQuest software (BD Biosciences, San Jose, CA, USA).

### 4.6. Elution assay

Same volume (5 µl/ml) of PACA and Dermabond® were added in RPMI medium and then collected elution mediums of PACA and Dermabond® for 24 hr. Control sample was not exposure with tissue adhesive. The cells were seeded in a 12 well plate and incubated for 24 hr. The cells were treated with elution mediums containing final 5% FBS. Following incubations for 24 hr, 48 hr and 72 hr, the cells were determined for cytotoxicity assessment by Cell counting-8 kit. OD_450_ was measured with a multi-microplate reader. The results of each sample were averaged and were expressed as a percentage of the control.

### 4.7. *In vivo* studies

Fourteen young male Sprague–Dawley rats (250–350 g) were purchased from Orient Bio Korea (Seoul, South Korea). The rats were housed in a controlled environment with temperatures of 22–24°C and a 12-hr light/dark cycle and fed commercial rat chow and water *ad libitum*. Adequate injection and anesthesia were performed to minimize pain or discomfort. All experimental animals were cared for and treated according to the “Guide for the Care and Use of Laboratory Animals” issued by the Korea University School of Medicine, Seoul, South Korea (IRB; KUIACUC-2012-139). All animals were anesthetized with inhaled isoflurane (1–5%) and then injected rompun 5–10 mg/kg and ketamine 30 mg/kg were intramuscularly. After anesthesia, hair from the animal's back was removed with electric clippers was applied for several minutes. The area was washed with wet cotton and dried with a gauze sponge. Two 2-cm long incisions were made on the back of each rat. The optimum PACA and Dermabond® were applied as a monolayer over the incision without additional treatment, according to instructions for the commercial tissue adhesive. Negative control group without an incision, and a positive control group was treated only 70% ethyl alcohol after the incision. Rats were sacrificed at day 7 after surgery to examine and compare the tissue reactions between the four groups. Each tissue specimen was fixed in 10% buffered formalin, embedded in paraffin, sectioned into sections 5 µm thick, and stained with H&E and MT to observe inflammatory cell infiltration and collagen distribution. The stained tissues were observed under an optical microscope.

### 4.8. Isolation of genomic DNA

The cells were incubated with PACA and Dermabond® for 24 hr at 37°C. Cells were harvested by trypsinization, washed once in PBS. DNA preparation was performed by High Pure PCR Template Preparation Kit (Indianapolis, IN, USA). Before starting the purification reaction, warm up the Elution buffer to 70°C. 200 µl of sample material was added 200 µl Binding buffer and 40 µl proteinase K. It was mixed immediately and incubated at 70°C for 10 min. After adding 100 µl isopropanol, the suspension was mixed properly and the sample was loaded into High filter tube. Centrifugation was performed at 8000 g for 1 min. After centrifugation, the High filter tube was combined with new collection tube and 500 µl of Inhibitor Removal buffer was added. Centrifugation was performed again at 8000 g for 1 min. Then the High filter tube was combined again with another new collection tube and washed two times with 500 µl of Wash buffer. After discarding the flow through liquid, the entire High pure assembly was centrifuged additional 10 sec at full speed and collection tube was discarded. Pre-warmed Elution buffer was added in order to elute the DNA and then centrifuged for 1 min at 8000 g. DNA concentration was calculated by the Nano-100 Micro-spectrophotometer (Allsheng, Hangzhou city, China).

### 4.9. Polymerase Chain Reaction (PCR)

To confirm the PCR products β-actin, amplified from same concentration of DNA samples, DNA fragment (776 bp) was generated by PCR from the genomic DNA using primers β-actin F (5′-ATGGGTCAGAAGGACTCCTACG-3′) and β-actin R (5′-AGTGGTACGACCAGAGGCATAC-3′) (Bioneer, Daejeon, South Korea). PCRs were conducted at 94°C (30 sec), 60°C (30 sec) and 72°C (1 min) for 27 cycles using the GeneAmp PCR system 9700 (applied biosystems, Foster City CA, USA). An aliquot of the reaction was separated on a 0.8% agarose gel and the intensity of signals was quantified with GelDoc2000 system (Bio-Rad, Hercules, CA, USA).

### 4.10. Raman spectroscopic measurements

For the Raman analysis, the cells were seeded in gold coated substrates and incubated for 24 hr. The cells were treated with PACA and Dermabond® (5 µl/10^5^ cells) and then further incubated for 24 hr. The cells were washed twice with PBS and fixed for 20 min in 4% paraformaldehyde in PBS at 4°C and washed with 5 ml PBS. To Raman analysis of DNA, DNA prepared with DNA preparation kit and measured DNA concentration using Nano-100 Micro-spectrophotometer. Same concentration of DNA samples were added in gold coated substrates. Raman spectra were acquired using the SENTERRA confocal Raman system (Bruker Optics Inc., Billerica, MA, USA) equipped with a 785 nm diode laser source (100 mW before objective) and the resolution of 3 cm^−1^. A 100× air objective (MPLN N. A. 0.9, Olympus), which produced a laser spot size of ∼1 µm was used to collect Raman signals and focus laser on a single cell. Raman spectra of cell and DNA were calculated as the average of fifteen measured. All Raman measurements are recorded with accumulation time of 60 sec in the 600–1750 cm^−1^ range, and spectral acquisitions were carried out using the OPUS software (Bruker, Optics Inc., Billerica, MA, USA).

### 4.11. Data analysis

PCA analysis was used to extract the major trends in each group. The determined principal vectors including Raman spectral intensities at 725 cm^−1^, 778 cm^−1^, 1002 cm^−1^, 1096 cm^−1^, 1257 cm^−1^ and 1656 cm^−1^ for cells and Raman spectral intensities at 759 cm^−1^, 799 cm^−1^, 911 cm^−1^, 1156 cm^−1^, 1256 cm^−1^, 1470 cm^−1^, and 1585 cm^−1^ for DNAs were used as the transfer function to determine the principal component (PC) scores for each spectrum of each group. Multi-SVM classifier with one-against-all approach based hierarchical structure was considered to be the three-class problem to classify the control, PACA and Dermabond® treated cells and DNAs. The performance of SVM classifier was assessed by the sensitivity (SEN), specificity (SPE), and accuracy (ACC), where SEN is used for evaluating the ability of the classifier to detect the cytotoxicity of cells and DNAs, SPE is for the ability of the classifier to specify control cells and DNAs and ACC is for the accuracy of the classifier. PCA-SVM algorithm was implemented with the MATLAB software (MathWorks Inc., Natick, MA, USA).

### 4.12. Statistics

Summary data were reported as mean ± SD. The results of WST and NR assays were obtained from eight sets of the same experiments. The results of LIVE/DEAD and TUNEL assays were obtained from three sets of the same experiments. Differences between each group were evaluated using the Mann-Whitney U test. P<0.05 was considered statistically significant.
